# The Association between Parenting Confidence and Later Child Mental Health in the Area Affected by the Fukushima Nuclear Disaster: The Fukushima Health Management Survey

**DOI:** 10.3390/ijerph19010476

**Published:** 2022-01-02

**Authors:** Rie Mizuki, Masaharu Maeda, Tomoyuki Kobayashi, Naoko Horikoshi, Mayumi Harigane, Shuntaro Itagaki, Hironori Nakano, Tetsuya Ohira, Hirooki Yabe, Seiji Yasumura, Kenji Kamiya

**Affiliations:** 1 Radiation Medical Science Center for Fukushima Health Management Survey, Fukushima Medical University, Fukushima 960-1295, Japan; masagen@fmu.ac.jp (M.M.); copepe@fmu.ac.jp (N.H.); harigane@fmu.ac.jp (M.H.); itasyun@fmu.ac.jp (S.I.); h-nakano@fmu.ac.jp (H.N.); teoohira@fmu.ac.jp (T.O.); hyabe@fmu.ac.jp (H.Y.); yasumura@fmu.ac.jp (S.Y.); kkamiya@hiroshima-u.ac.jp (K.K.); 2 Department of Disaster Psychiatry, School of Medicine, Fukushima Medical University, Fukushima 960-1295, Japan; tomokoba@fmu.ac.jp; 3 Department of Public Health, School of Medicine, Fukushima Medical University, Fukushima 960-1295, Japan; 4 Department of Neuropsychiatry, School of Medicine, Fukushima Medical University, Fukushima 960-1295, Japan; 5 Department of Epidemiology, School of Medicine, Fukushima Medical University, Fukushima 960-1295, Japan; 6 Research Institute for Radiation Biology and Medicine, Hiroshima University, Fukushima 960-1295, Japan

**Keywords:** parenting confidence, child mental health, SDQ, nuclear disaster

## Abstract

After the 2011 Fukushima Daiichi Nuclear Power Station accident, the Fukushima Health Management Survey was conducted to assess children’s lifestyle and mental health conditions. The participants in this study were 1126 children, aged 0 to 3 years, living in the evacuation zone at the time of the disaster. The parenting confidence of their mothers was assessed using a self-administered questionnaire as a baseline in 2013. We examined the association of parenting confidence level at baseline, using a total difficulty score of the Strengths and Difficulties Questionnaire (SDQ) and reluctance to attend school among children in a follow-up study in 2016 and 2017. As a result, no confidence was reported by 178 (15.8%) mothers, while 477 (42.4%) responded with “not sure” and 471 (41.8%) were confident. In the multiple logistic analysis, after adjusting for covariates such as the child’s sex, age, and current health condition, the group lacking parenting confidence demonstrated a significantly higher risk level for SDQ total difficulties (OR, 2.8; 95% CI, 1.59–4.93) and reluctance to attend school (OR = 1.98, 95% CI: 1.24–3.18) than the confident mothers. After a major disaster, which can have long-term effects on communities, intensive psychological care for mothers with young children is needed to prevent various mental health problems in their children.

## 1. Introduction

Disasters make parenting practices difficult by changing living environments, such as through home damage and lack of utilities, and by deteriorating family functioning, which is characterized by disrupted family communications [1,2,3] and poor parenting [4]. Reduced parenting quality after a disaster, which can be characterized by insufficient attention, support, and care for children [5], was identified as a major risk factor for post-traumatic stress disorder (PTSD) in children, as a result of various types of natural and man-made disasters [6]. Conversely, when mothers’ functioning is sustained at a higher level, it buffers the negative impacts of disasters on child mental health [7]. The importance of the link between parental conditions and children’s mental health after a disaster indicates that the retention and reinforcement of parental functioning is a critical component of the well-being of children.

After the Fukushima nuclear disaster, many residents were evacuated, both involuntarily and voluntarily. Two years after the disaster, 20–40% of families who had been evacuated by a governmental order were still separated from family members with whom they had lived [8,9]. Several families with children chose to evacuate only mothers and children voluntarily, while leaving the fathers at home to avoid loss of income [10,11]. In addition to family separation, evacuation deprived mothers of supportive resources, burdening them with the increased stress of raising children while adapting to a new environment. Evacuated mothers reported feeling isolated and struggled with the increased burden of child rearing, as the people on whom they typically relied for childcare were no longer available [12,13]. Not surprisingly, evacuation increased the complexity of raising children by destabilizing and complicating the living environments of these families, including mothers’ emotional conditions [14]; moreover, evacuation may have led to the deterioration of children’s mental health, as shown by higher rates of mental health problems in the children from the evacuation zones [15].

Parenting confidence is defined as caregivers’ perception of their ability to care for and understand their children, and is one of the key factors in healthy parent–child relationships [16]. Higher maternal confidence is associated with the healthy development of their children, both physically and mentally, including children’s cognitive ability, motor skills, and autonomic stability, as well as superior adaptive behaviors and fewer disruptive behaviors [17,18,19,20]. Caregivers’ confidence influences their parenting practices. Mothers who reported a lack of confidence in raising their child tended to employ dysfunctional or poor parenting practices [21,22], which could be associated with various child psychological maladaptations, including externalizing problems [23], attention deficit hyperactivity disorder [24], and conduct problems [25]. Hence, lower parenting confidence has been linked to child mental health problems due to dysfunctional parenting practices [26,27].

Parenting confidence was found to be influenced by parental mental health conditions. Lower parenting confidence is associated with depressive symptoms in postpartum mothers [28,29] and in mothers with serious mental illnesses, such as bipolar disorder and schizophrenia [30]. After disasters, many Japanese mothers with young children reported difficulties with child-rearing [31]. They also reported a sense of guilt derived from raising children with unstable mental conditions and the inability to manage their children [32]. Furthermore, declined parenting confidence and its connection with poor mental health conditions were identified among mothers in Fukushima [33].

Despite some research that investigated parental conditions including the deterioration of parenting confidence [34] and child mental health [15] after the Fukushima nuclear disasters, to the best of our knowledge, only a small number of studies utilized a longitudinal design to prospectively examine the association between parenting confidence and child mental health problems that emerged several years later. As the nuclear disaster exerted long-term effects on the mental health of parents and children [35], it is important to empirically reveal its lasting impact on child mental health. Thus, in this study, we examined how maternal parenting confidence during the first three years of their child’s life influenced mental health conditions in their children three or four years later in people affected by the Fukushima nuclear disaster by using a longitudinal research design. We hypothesized that a lack of parenting confidence in mothers would predict greater mental health problems several years later, such that children with unconfident mothers in 2013 would be more likely to experience emotional and behavioral issues at the clinical level three or four years later. We also hypothesized that mothers’ lack of parenting confidence would be associated with their children’s school attendance problems, and that the children of unconfident mothers would exhibit reluctance to attend school three or four years later.

## 2. Materials and Methods

### 2.1. Study Design

The Mental Health and Lifestyle Survey is a part of the Fukushima Health Management Survey, which seeks to assess the mental and physical health conditions of the residents affected by the nuclear disaster, as well as to provide necessary care. The details of the survey have been explained elsewhere [36]. The target area encompassed ten municipalities from which all residents were evacuated, as well as three municipalities from which residents in selected areas were evacuated: Hirono, Naraha, Tomioka, Kawauchi, Okuma, Futaba, Namie, Katsurao, Iitate, Minamisoma, Tamura, and part of Date City.

A self-administered questionnaire packet was mailed to each member of the target population from the aforementioned area every year from 2012. The filled questionnaires were returned via mail to the research team. The current longitudinal study defined the data collection in 2013 as the baseline and the data collected in 2016 and 2017 as the follow-up.

### 2.2. Participants

The target population of the current study was a total of 4625 children aged zero to three years as of 2 April 2012, and living in one of the 13 municipalities in the coastal area of Fukushima, which were the targets of the Mental Health and Lifestyle Survey. 

**Figure 1:**
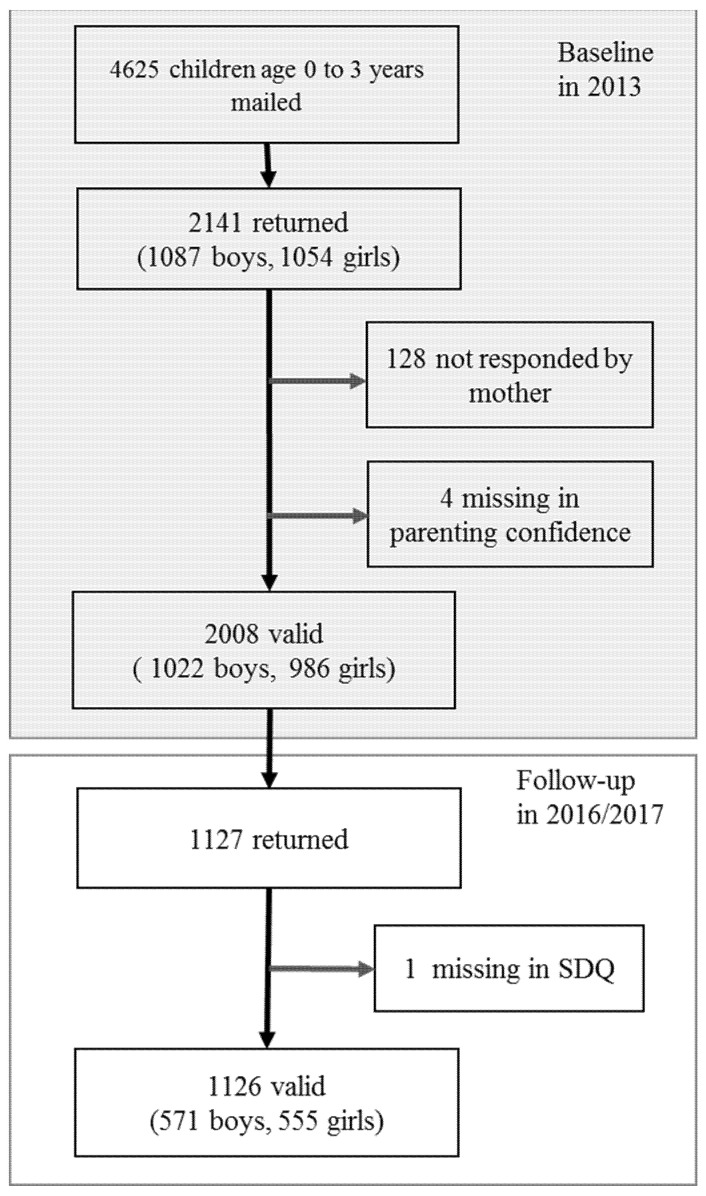
Flow chart of study participants.

The baseline data in the current study were collected in 2013. A questionnaire packet was mailed to the target population, and a total of 2143 parents/guardians filled and returned the questionnaires (response rate: 46.3%). Among these, 132 questionnaires were not completed by mothers and 3 participants did not provide an answer to the item on parenting confidence, resulting in a total of 2008 valid responses. A follow-up study was conducted in 2016 and 2017 by mailing a questionnaire packet to the target sample again to assess the mental health conditions of the children. Of the participants with valid responses at baseline, 1127 returned the questionnaire packet in the follow-up study (follow-up rate: 52.6%), about three or four years after the baseline. Excluding one case with missing data on child mental health, 1126 cases were included in the statistical analyses. The flow chart of the sample is shown in Figure 1.

### 2.3. Measures

#### 2.3.1. Baseline Assessment

At the baseline (2013), we inquired about parenting confidence as a predictor and the participants’ characteristics as covariates. Parenting confidence was assessed through one item asking, “are there any moments when you don’t feel confident about child rearing?” The participants answered with either “yes,” “no,” or “not sure.” The original question asked about the absence of parenting confidence; the participants who did not feel confident in child-rearing answered “yes,” whereas those who were confident selected “no.” Those who could not decide between “yes” and “no” marked “not sure.” The validity of this question item was indicated by its association with parenting and general self-efficacy [21]. To simplify the interpretation of the analyses and results, we reversed the coding of the answers so that their responses corresponded with the presence of their confidence in parenting (i.e., “yes” = confident, “no” = not confident), while the “not sure” response remained as it was. In addition, the characteristics of the participants, such as the child’s age, sex, stunting, current health condition, sleep duration, hospitalization history, and evacuation location were collected as covariates at baseline. Stunting was defined as either a height or weight of two standard deviations or more below the child growth standards [37]. Evacuation location was defined as the residential location either outside of or in the Fukushima prefecture in 2013.

#### 2.3.2. Follow-Up Assessment

In the follow-up (2016 or 2017), the mental health conditions of the children were defined as the main outcome, measured using the parent version of the Strengths and Difficulties Questionnaire (SDQ) [38] and a question on reluctance in going to school. The SDQ consists of 25 items, in which parents/caregivers report on how their child has been during the six previous months, assessing the psychopathology as well as the positive strengths of children from the age of four to sixteen years. The respondents answered on a 3 point Likert scale (0 = not true, 1 = somewhat true, 2 = definitely true). The questionnaire comprised five subscales: (1) emotional symptoms, which reflect anxiety and mood problems (e.g., “many fears, easily scared”); (2) conduct problems, which tap into behavior issues, such as tantrums and misbehavior (e.g., “often lies or cheats”); (3) hyperactivity/inattention, which describe overactive tendency and attention problems (i.e., “easily distracted, concentration wanders”); (4) peer relationship problems, which include items regarding interactions and relationships with other children (e.g., “rather solitary, prefers to play alone”); and (5) prosocial behavior, which assesses positive social behaviors (e.g., “often offers to help others”). Each subscale contains five question items, and each subscale’s score ranges from 0 to 10. 

The total difficulty score was calculated by summing the scores for emotional symptoms, conduct problems, hyperactivity/inattention, and peer problems. The scores ranged from 0 to 40, based on 20 items. The high-risk groups that fell in the clinical ranges were defined by the respective cutoff scores of total difficulties and each subscale’s score (total difficulties ≥16, emotional symptoms >5, conduct problems >5, hyperactivity-inattention >7, peer problems >5, and prosocial behavior <5). The validity and internal consistency of the Japanese version of the SDQ have been demonstrated [39,40]. In addition, the test-retest reliability was good except for the peer problems scale: 0.82 for total difficulties, 0.77 for emotional symptoms, 0.73 for conduct problems, 0.77 for hyperactivity, 0.58 for peer problems, and 0.78 for prosocial behavior [41]. The participating children were aged 4–7 years when their mothers completed the SDQ. Moreover, given the validated target age range of the measure, SDQ was not administered at baseline when the participating children were aged 0–3 years. 

Children’s reluctance to attend school was assessed as another index of children’s mental health conditions in 2016 and 2017. One question item asked, “Has your child ever expressed reluctance in going to school?” Mothers with children who were below the age of elementary school were asked about their child’s attendance at kindergarten or daycare. The participants answered with either “no,” “yes,” or “not enrolled in kindergarten or daycare.” Mothers with elementary-school-aged children answered with either “no” or “yes.” While “no” and “yes” were coded as 0 and 1 respectively, the cases with the answer “not enrolled in kindergarten or daycare” were coded as missing.

### 2.4. Ethics

The protocol of the current study was approved by the Ethics Committee of Fukushima Medical University (No. 2020–239). A written explanation of the study protocol comprising the following was provided to the participants: (1) the voluntary nature of participation, (2) option to withdraw from the study at any time, (3) no disadvantage associated with refusal of or withdrawal from participation, and (4) the protection of the participants’ identifiable data. Returning a questionnaire was considered consent for participation in the study. The dataset used in the current study did not contain any identifiable information.

### 2.5. Statistical Analysis

First, we categorized the participants into two groups based on the SDQ total difficulties scores (i.e., high-risk group ≥ 16; low-risk group < 16) measured in the follow-up of 2016/17 and compared the means and frequencies of the demographic characteristics assessed in the baseline of 2013 between the groups using *t*-test or chi-square test. Second, logistic regression analyses were performed between parenting confidence in 2013 and SDQ total difficulty as well as reluctance to attend school in 2016/17, with adjustment for children’s age and sex. Third, we conducted multiple logistic regression analyses of parenting confidence in 2013 on SDQ total difficulty and reluctance to attend school in 2016/17, adjusted for child’s sex, age, stunting, current health condition, sleep duration, hospitalization history, and evacuation location. Fourth, multiple logistic regression analyses for the five SDQ subscales in 2016/17 were conducted with adjustment for the same covariates. For missing values, we used listwise deletion, which resulted in varied sample sizes for different statistical analyses. 

All the statistical analyses were conducted using IBM SPSS Statistics for Windows (version 26.0; IBM Corp, Armonk, NY, USA). The level of statistical significance was set at *p* < 0.05 (two-tailed).

## 3. Results

The participants’ characteristics are summarized in Table 1. Among a total of 1126 mothers, 107 (9.5%) mothers had children categorized in the SDQ high-risk group (SDQ total difficulties score ≥16), while 1019 (90.5%) mothers had children in the SDQ low-risk group. Regarding parenting confidence, in the SDQ high-risk group, 26.2%, 45.8%, and 28.0% reported confidence, not sure, and not confident, respectively; meanwhile, 43.5%, 42.0%, and 14.5% were found in the SDQ low-risk group, respectively. The high-risk group included significantly more mothers who reported not being confident (28.0% vs. 14.5%) and fewer mothers who reported being confident (26.2% vs. 43.5%) than the low-risk group (*χ*^2^ = 18.4, *df* = 2, *p* < 0.001). Significantly more children in the SDQ high-risk group were rated poorly (poor or very poor: 2.8% vs. 1.7%; *χ*^2^ = 9.83, *df* = 2, *p =* 0.007) for their health conditions and had been hospitalized (32.7% vs. 23.7%; *χ*^2^ = 4.25, *df* =1, *p* = 0.039) compared to the low-risk group. In addition, the children in the high-risk group were more likely to be reluctant to attend school (*n* = 34, 31.8%) than those in the low-risk group (*n* = 134, 13.2%; *χ*^2^ = 26.17, *df* = 1, *p* < 0.001). No significant differences were found in children’s age, sex, stunting, and evacuation location.

The results of the multiple logistic regressions for SDQ total difficulties and reluctance to attend school are shown in Table 2. In the model with adjustment for children’s age and sex, the children of mothers lacking parenting confidence (*OR* = 3.24, 95% CI: 1.87–5.62) and unsure about their confidence (*OR* = 1.81, 95% CI: 1.11–2.93) displayed a significantly higher risk for emotional and behavioral problems. After adjusting for the child’s age, sex, stunted current health condition, sleep duration, hospitalization history, and evacuation location, the multivariable-adjusted ORs of non-confident mothers (*OR* = 2.75, 95% CI: 1.56–4.84) and not-sure mothers (*OR* = 1.65, 95% CI: 1.01–2.70) for the SDQ high-risk group remained significantly higher than those of confident mothers. These results were consistent with our hypothesis that a lack of parenting confidence in mothers would be associated with a high risk of emotional and behavioral problems in children at the clinical level three or four years later.

For reluctance to attend school, our hypothesis that mothers’ lack of parenting confidence would predict their children’s school attendance problems was also supported. In the model adjusted for the children’s age and sex, the children of unconfident mothers and mothers who were not sure demonstrated significantly higher multivariable-adjusted ORs of 2.24 (95% CI: 1.42–3.54) and 1.50 (95% CI: 1.02–2.19) for the risk of reluctance to attend, compared to those of confident mothers. After adjusting for all the covariates, only the multivariable-adjusted OR of not confident mothers (*OR* = 1.99, 95% CI: 1.24–3.20) remained significantly higher compared to those of confident mothers. Mothers who reported not being confident at parenting in 2013 were more likely to have children suffering from mental and behavioral issues measured using SDQ as well as reluctance to attend school three or four years later, even after ruling out the effect of the child’s age, sex, stunting, current health condition, sleep duration, hospitalization history, and evacuation location. In addition, mothers who were unsure about their parenting in 2013 were at a higher risk of mental health problems in their children than confident mothers several years later.

The results of the multiple logistic analyses of the five SDQ subscales are presented in Table 3. After adjusting for all the covariates, the children of unconfident mothers presented significantly higher ORs compared to confident mothers: 2.53 (95% CI: 1.33–4.82) for emotional symptoms, 4.50 (95% CI: 2.39–8.48) for conduct problems, 1.91 (95% CI: 1.11–3.28) for hyperactivity/inattention, and 3.26 (95% CI: 1.48–7.17) for peer relationship problems. Additionally, the children of mothers who were not sure about their parenting confidence presented a significantly higher OR of 2.58 (95% CI: 1.52–4.39) for emotional symptoms and 2.23 (95% CI: 1.11–4.48) for peer relationship problems, compared to those of confident mothers. For emotional symptoms, the OR of the children of mothers who were not sure was higher than that of the non-confident group. No significant association was found between parenting confidence and prosocial behavior.

Children whose mothers lacked confidence in parenting during their first three years had almost two- to four-fold risks of developing emotional symptoms, conduct problems, hyperactivity/inattention, and peer relationship problems when they reached the age range of four to seven years, compared to the children of confident mothers. Not only lack of confidence but also maternal uncertainty about parenting confidence during the first three years of the child’s life predicted significantly higher risks of emotional symptoms and peer relationship problems in children several years later.

## 4. Discussion

This is the first longitudinal study to examine the long-term impact of maternal parenting confidence after the Fukushima nuclear disaster on the development of mental health issues among children. The lack of parenting confidence in mothers at the baseline (two years after the disaster) was associated with various mental health problems in their children, including mental health problems measured using SDQ and reluctance to attend school, at the follow-up (several years later). 

The sum of the ratios of mothers who reported either a lack of confidence or uncertainty regarding parenting confidence was 58.2%. This was similar to the findings regarding mothers with 18 month old children in Fukushima (50% in 2010, 60% in 2011 and 53% in 2012; [34]). On the other hand, research involving the mothers of 3–4 month old infants from Fukushima found that only 22% of mothers lacked parenting confidence in 2007 [21]. A substantial difference in the ratios of parenting confidence was found in these two studies, although both studies involved participants from the Fukushima prefecture. This sizable difference may be related to the timing of the measurement of parenting confidence. One of the contributing factors may have been the developmental stage of the child in the samples of these two studies: 3–4 month old and 18 month old infants. As children more frequently express negative emotionality during the second year of life [42], parents also face intensified parenting stress [43] and increased parenting hassles [44]. Compared to taking care of a 3–4 month-old infant, raising a toddler or an older child can become increasingly complicated. The age of the child may have resulted in a higher ratio of lack of parenting confidence in the current study, as the majority (67.4%) of the participants were raising a child aged two or three years.

The nuclear disaster could be another factor that may have elevated the number of mothers who were not confident. Goto and Rudd [34] found a significant increase in lack of parenting confidence (i.e., not sure and not confident) from 2010 (50%) to 2011 (60%) and a decrease to a non-significant level from 2011 to 2012 (53%). The percentage of mothers who reported being unsure or not confident in the current study was 58.2%, which was similar to the post-disaster ratios, indicating that the parenting confidence in our sample was likely to have been inflated by the nuclear disaster. In particular, it is noteworthy that the lack of confidence in the current study measured two years after the disaster is higher than the ratio measured a year after the disaster by Goto and Rudd [34]. This could be attributed to the fact that the current study sample comprised residents from the evacuation zone, who were most extensively affected by the disaster. Thus, the finding that 58.2% of mothers lacked confidence in their own parenting even two years after the disaster may reflect the prolonged and severe negative impact of the nuclear disaster on maternal confidence. 

Several previous studies reported the influence of mothers’ subjective perceptions of their own parenting, such as parenting confidence or self-efficacy, on their child’s psychological conditions, such as emotionality, frustration tolerance, and peer social skills [26,45,46]. The current findings show that a lack of parenting confidence during the first three years of a child’s life had a long-term effect on the mental health of the child, as measured using the SDQ total difficulties score. While some of these past studies feature samples with difficult life experiences, such as homelessness and immigration, a similar tendency was found in this study, which investigated participants confronted with severely stressful and unstable life conditions after the nuclear disaster. While the past findings were based on cross-sectional studies and implications for causal relationships were limited, the longitudinal design of the current study could show that parenting confidence during the first three years of the child’s life predicted the child’s mental health three or four years later. The current study could not directly examine the effect of the nuclear disaster on maternal mental health conditions or maternal parenting confidence, as previous research has revealed the deteriorating impact of nuclear disasters on mental health in mothers [47,48] and the association between lack of parenting confidence and mental health problems in mothers [29]. The findings from the current longitudinal study further add to the research finding that maternal parenting confidence—diminished specifically by the nuclear disaster—may predict the development of mental health problems in their children several years later. This prediction suggests a shift of focus from children with mental health problems to their mothers, particularly at the early stage of children’s lives. Proactive interventions aimed at mothers may be crucial when considering treatment for children with mental health problems after nuclear disasters. 

The current findings also support our prediction that the children of mothers who were not confident would be at a higher risk of reluctance to attend school several years later. In Japan, the issue of school non-attendance has grown over the past decades and has drawn attention from society [49]. School adjustment has become a serious issue for children from the areas affected by the Fukushima nuclear disaster [50], as indicated by reports of severe bullying of students evacuated from Fukushima [51]. The mental health issues of children directly affected by the Fukushima disaster may manifest in their adaptation to school life, which is congruent with the our results showing SDQ peer relationship problems as well as increased mental health problems in school-aged children who were evacuated from Fukushima [52]. The current findings—which indicate a link between parenting confidence and children’s school attendance problems years later—could have some implications for understanding and preventing delayed school-related behavioral issues among affected children.

When mothers do not maintain a sufficient sense of security—including a secure living environment and financial stability, information on the health risks of radiation, and social support from the family and community members—they may not capitalize on their parenting competence due to their own struggle with recovery from the nuclear disaster. Therefore, such mothers may likely fail to provide emotional availability and sensitive care practices to their children, which are related to their loss of confidence in parenting [46]. Subsequently, mothers can develop negative perceptions of their own parenting, failure to provide quality care, and mental and behavioral difficulties in their child; similar struggles have been reported by mothers in Fukushima [53,54]. Under ordinary circumstances, the members of local communities, including family members, friends, and daycare/kindergarten staff, notice the plight of these mothers and make arrangements for appropriate interventions or support to facilitate child rearing. Unfortunately, the availability of such support for mothers during the aftermath of the nuclear disaster is assumed to have been drastically reduced because of the focus on evacuation, survival, and immediate recovery in the affected areas [11,55]. Even though support services are available in evacuation destinations, mothers relocating there may require a considerable amount of time to become familiar with new community cultures and to utilize local services for their children. 

A lack of confidence in parenting was associated with increased ORs of emotional symptoms, conduct problems, hyperactivity/inattention, and peer relationship problems. The current findings are consistent with those of past research indicating maternal self-efficacy as a significant predictor of externalizing problems in children, which comprises conduct problems and hyperactivity/inattention [56]. Given the link between lower parenting confidence and poor parenting competence [21,22], some mothers lacking parenting confidence could not help resorting to harsh parenting (e.g., corporal punishment) as an attempt to manage their children’s behavior, while coping with the increased stress and limited resources derived from evacuation due to the Fukushima nuclear disaster.

Moreover, higher ORs of internalizing problems (i.e., emotional symptoms and peer relationship problems) were aligned with previous research on Japanese samples; maternal parenting stress is related to emotional symptoms and peer relationship problems in their children [57]. Unlike externalizing problems, higher risks for internalizing problems were present not only in mothers lacking confidence, but also in mothers who were not sure about their parenting. In contrast to the effects of tsunami disasters, concerns for health risks caused by a nuclear disaster could last over several decades and inflate anxiety, especially over radiation exposure. The aftereffects of the Fukushima nuclear disaster could make many mothers, who otherwise felt confident, resort to anxious parenting, which could be related to internalizing problems [4]. 

One of the advantages of measuring parenting confidence is that improvements in parents’ perception of their own parenting have been achieved through interventions. After participation in short-term parenting training programs, parenting self-efficacy was enhanced in mothers with infants [58] and in various caregivers (for a review, see [59]). For the link between knowledge of child rearing and confidence [19], even a parenting workshop that educates struggling parents regarding basic knowledge of parenting may benefit children during the aftermath of a disaster.

Although financial assistance was provided by the national and/or local governments after the Fukushima disaster in 2011, evacuated parents claimed that this financial/economic support did not fulfill their needs [10,11]. As a form of post-disaster support, the alleviation of financial constraints has been suggested to protect child mental health by promoting healthy psychological status and parenting efficacy in mothers [60]. Therefore, financial support that specifically targets families with young children should be considered in future disaster responses.

The unique findings of this study were the link between mothers who were unsure about their parenting and the increased risk of child mental health problems several years later. Specifically, significant effects were found for total difficulties, emotional symptoms, and peer relationship problems. Particularly for emotional symptoms and peer relationship problems, the ORs of the mothers who answered with “not sure” were higher than those of the unconfident mothers, suggesting that the “not sure” response does not only represent another expression of lack of confidence but also connotes a unique effect. For instance, mothers who reported a lack of confidence may be more likely to seek help from social resources for child rearing than mothers who are not sure about their parenting, leading to better management of their child’s mental health issues despite their lower parenting confidence. 

Taking into consideration the points described above, the following recommendations are suggested: (1) asking about parenting confidence, (2) arranging parenting training workshops, and (3) providing financial assistance for families with young children. First, during home visits or health check-ups for children, asking mothers of children aged 0–3 years about their parenting confidence can be a standardized instrument integrated into routine assessment procedures for maternal and child health clinicians. Subsequently, based on the assessment, parenting training, involving basic knowledge of child development and specific skills of children, should be conducted, targeting mothers who are not sure about their own confidence, in addition to mothers who lack parenting confidence. Additionally, information on childcare resources in the community should be provided to these mothers. Lastly, after a nuclear disaster, it is important to provide financial assistance, specifically to families with young children to meet their unique needs, including larger housing for families with young children, arrangements for daycare, transportation fees (allowing frequent visits between separated family members), and obtaining age-appropriate clothes and hygiene products for developing children. These interventions, which focus on improving conditions where mothers can feel more secure about their child rearing, should be integrated into recovery programs for nuclear disasters, specifically for the prevention of child mental health issues.

This study featured several limitations. First, the sample was not representative due to the lower response and follow-up rates. In order to contribute to the health of the participants and to maintain the response rate, the individual summary of survey responses was sent to each participant who had submitted the questionnaire since 2015. Despite this effort, the response rate decreased from 46.3% in 2013 to 28.1% in 2016 and 21.2% in 2017 [61]. The decline in participation in the survey could be attributed to the fact that most of the target population was too busy to take time to complete the questionnaire due to unstable evacuation living conditions. In addition, since the survey was funded by the Fukushima prefectural government, a distrust of authorities [62] might have been partially responsible for the decreasing response rates. This calls for careful interpretation of the study findings. Second, the reliability of the measure for parenting confidence was not indicated; this reliability (e.g., test-retest reliability) must be examined in future research. Third, the potential confounding factors that may have affected parenting confidence and child mental health, such as parental socioeconomic status, maternal mental health conditions, child’s birth order, anthropometrics at birth, and family composition, were not adjusted. Fourth, the assessment of the child mental health conditions relied solely on the mothers’ reports. Taking into consideration the bias associated with maternal mental health in reporting child mental health conditions, the current study incorporated reluctance in going to school as another index of child mental health, which represented a less subtle and more observable behavior. Nonetheless, the use of objective measures, including behavior observation by and reports from other informants, such as teachers or researchers, is crucial for future research to reinforce the validity of the current findings. 

Despite these research constraints, which may be inevitable in post-disaster conditions, several clinical and policy implications are raised by the current study. Parenting confidence, which can be assessed using one question, may be useful for predicting long-term mental health problems in children. During the aftermath of a nuclear disaster, professionals involved in the field of mental and child health, particularly social service workers and public health nurses from local governments, may use maternal parenting confidence as a screening tool for high-risk families when the assessment of child conditions is limited or difficult. Additionally, not only mothers lacking parenting confidence but also those who express uncertainty about parenting confidence may benefit from support services for themselves and their children. It is assumed that proactively providing support may prevent the development or deterioration of mental health problems in children over several subsequent years.

## 5. Conclusions

In summary, this study found a significant association between mothers’ parenting confidence after the Fukushima nuclear disaster and their children’s emotional and behavioral problems and school attendance problems occurring several years later. In the aftermath of a nuclear disaster, the assessment of parenting confidence may be a useful tool for predicting long-term mental health problems in children, providing mothers who lack or are uncertain about parenting confidence with proactive interventions as preventative measures for their children’s mental health problems. 

## Figures and Tables

**Table 1:** Characteristics of the participants (*n* = 1126).

		SDQ < 16 (*n* = 1019)		SDQ ≥ 16 (*n* = 107)		*p*	Cramer’s v/ Cohen’s d
		n/Mean	%/SD	n/Mean	%/SD		
Parenting confidence	Confident	443	43.5	28	26.2	<0.001	0.128
	Not sure	428	42.0	49	45.8		
	Not confident	148	14.5	30	28.0		
Age, years	Mean	2.0	0.86	1.9	0.82	0.167	0.141
	0	24	2.4	3	2.8	0.239	0.061
	1	306	30.0	34	31.8		
	2	336	33.0	43	40.2		
	3	353	34.6	27	25.2		
Sex	Girl	525	51.5	46	43.0	0.093	0.050
	Boy	494	48.5	61	57.0		
Stunting	No	988	97.0	99	92.5	0.026 ^†^	0.011
	Yes	31	3.0	8	7.5		
Current health conditions	Very good or good	697	69.1	58	54.2	0.007 ^†^	0.094
	Fair	295	29.2	46	43.0		
	Poor or very poor	17	1.7	3	2.8		
Sleep duration, hours	Mean	10.0	0.83	9.8	0.88	0.025	0.229
Hospitalization history	No	776	76.3	72	67.3	0.039	0.061
	Yes	241	23.7	35	32.7		
Evacuation location	Inside of Fukushima	659	64.7	65	60.7	0.420	0.024
	Out of Fukushima	360	35.3	42	39.3		
Reluctance to attend school	No	880	86.8	73	68.2	<0.001	0.153
	Yes	134	13.2	34	31.8		

Note. All the characteristics were measured in 2013, except for reluctance in going to school, which was measured in 2016/2017. *p* for t-test or chi-square test. ^†^ Fisher’s exact test.

**Table 2:** Odds ratio of multiple logistic regression analyses for the high-risk group of SDQ and reluctance to go to school.

		SDQ Total Difficulties (*n* = 1110)						Reluctance to Attend School (*n* = 1105)					
		Age and Sex-Adjusted			Multiple *			Age and Sex-Adjusted			Multiple *		
		OR	CI Lower	CI Upper	OR	CI Lower	CI Upper	OR	CI Lower	CI Upper	OR	CI Lower	CI Upper
Confidence in parenting													
	Yes	ref.			ref.						ref.		
	Not sure	**1.81**	1.11	2.93	**1.65**	1.01	2.70	**1.50**	1.02	2.19	1.45	0.99	2.14
	No	**3.24**	1.87	5.62	**2.75**	1.56	4.84	**2.24**	1.42	3.54	**1.99**	1.24	3.20
Age		0.83	0.657	1.048	**0.78**	0.61	0.99	**0.68**	0.56	0.83	**0.66**	0.54	0.80
Sex		1.383	0.921	2.077	1.33	0.88	2.02	1.12	0.80	1.56	1.14	0.81	1.61
Stunting					1.34	0.50	3.61				1.70	0.78	3.69
Current health condition					**1.51**	1.04	2.19				**1.47**	1.07	2.02
Sleep duration					**0.75**	0.59	0.97				1.07	0.87	1.31
Hospitalization history					1.32	0.84	2.08				1.16	0.79	1.71
Evacuation out of Fukushima					1.21	0.78	1.87				1.36	0.95	1.93

Note. SDQ = Strengths and Difficulties Questionnaire. Statistically significant in bold. Sex: 1 = girls, 2 = boys. Current health condition: 1 = very good or good, 2 = fair, 3 = bad or very bad. Hospitalization history: 1 = no, 2 = yes. Evacuation: 1 = in Fukushima, 2 = out of Fukushima. Stunting: 0 = no, 1 = yes. Statistical significance are in bold. * Adjusted for age, sex, stunting, current health condition, sleep duration, hospitalization history, and evacuation out of Fukushima.

**Table 3:** Odds ratio of multiple logistic regression analyses for the high-risk group of SDQ subscales (*n* = 1110).

	Emotional Symptoms			Conduct Problems			Hyperactivity/Inattention			Peer Relationship Problems			Prosocial Behavior		
	OR	CI Lower	CI Upper	OR	CI Lower	CI Upper	OR	CI Lower	CI Upper	OR	CI Lower	CI Upper	OR	CI Lower	CI Upper
Parenting confidence															
Confident	ref.			ref.			ref.			ref.			ref.		
Not sure	**2.58**	1.52	4.39	1.38	0.74	2.56	0.97	0.61	1.55	**2.23**	1.11	4.48	0.94	0.65	1.36
Not confident	**2.53**	1.33	4.82	**4.50**	2.39	8.48	**1.91**	1.11	3.28	**3.26**	1.48	7.17	1.45	0.91	2.30
Age	1.03	0.80	1.33	**0.66**	0.50	0.88	**0.74**	0.59	0.94	0.85	0.62	1.16	**0.75**	0.62	0.91
Sex	0.91	0.59	1.40	1.09	0.66	1.78	1.49	0.99	2.26	1.46	0.83	2.56	**2.24**	1.58	3.16
Stunting	1.45	0.54	3.87	0.76	0.17	3.30	1.69	0.68	4.21	1.66	0.48	5.75	1.38	0.59	3.22
Current health condition	1.29	0.87	1.93	1.03	0.65	1.64	0.94	0.63	1.41	1.47	0.90	2.39	1.24	0.91	1.71
Sleep duration, hours	1.01	0.78	1.32	1.03	0.77	1.38	0.82	0.64	1.05	0.91	0.66	1.27	0.92	0.75	1.13
Hospitalization history	0.92	0.55	1.53	1.30	0.75	2.25	1.35	0.86	2.12	1.25	0.69	2.29	0.90	0.61	1.33
Evacuation location	1.66	1.07	2.58	0.92	0.55	1.56	0.80	0.51	1.25	1.24	0.70	2.20	0.88	0.62	1.26

Note. SDQ = Strengths and Difficulties Questionnaire. Sex: 1 = girls, 2 = boys. Current health condition: 1 = very good or good, 2 = fair, 3 = bad or very bad. Hospitalization history: 1 = no, 2 = yes. Evacuation: 1 = in Fukushima, 2 = out of Fukushima. Stunting: 0 = no, 1 = yes. Statistical significances are in bold. All multiple regression analyses were adjusted for sex, age, stunting, current health conditions, sleep duration, hospitalization history, and evacuation from Fukushima.

## Data Availability

The data are not publicly available due to ethical considerations.
